# The toxin–antitoxin RNA guards of CRISPR-Cas evolved high specificity through repeat degeneration

**DOI:** 10.1093/nar/gkac712

**Published:** 2022-08-26

**Authors:** Feiyue Cheng, Aici Wu, Chao Liu, Xifeng Cao, Rui Wang, Xian Shu, Lingyun Wang, Yihan Zhang, Hua Xiang, Ming Li

**Affiliations:** CAS Key Laboratory of Microbial Physiological and Metabolic Engineering, State Key Laboratory of Microbial Resources, Institute of Microbiology, Chinese Academy of Sciences, Beijing, China; CAS Key Laboratory of Microbial Physiological and Metabolic Engineering, State Key Laboratory of Microbial Resources, Institute of Microbiology, Chinese Academy of Sciences, Beijing, China; College of Life Science, University of Chinese Academy of Sciences, Beijing, China; CAS Key Laboratory of Microbial Physiological and Metabolic Engineering, State Key Laboratory of Microbial Resources, Institute of Microbiology, Chinese Academy of Sciences, Beijing, China; College of Life Science, University of Chinese Academy of Sciences, Beijing, China; School of life Sciences, Hebei University, Baoding, Hebei, China; CAS Key Laboratory of Microbial Physiological and Metabolic Engineering, State Key Laboratory of Microbial Resources, Institute of Microbiology, Chinese Academy of Sciences, Beijing, China; CAS Key Laboratory of Microbial Physiological and Metabolic Engineering, State Key Laboratory of Microbial Resources, Institute of Microbiology, Chinese Academy of Sciences, Beijing, China; College of Life Science, University of Chinese Academy of Sciences, Beijing, China; CAS Key Laboratory of Microbial Physiological and Metabolic Engineering, State Key Laboratory of Microbial Resources, Institute of Microbiology, Chinese Academy of Sciences, Beijing, China; College of Plant Protection, Shandong Agricultural University, Taian, Shandong, China; School of life Sciences, Hebei University, Baoding, Hebei, China; CAS Key Laboratory of Microbial Physiological and Metabolic Engineering, State Key Laboratory of Microbial Resources, Institute of Microbiology, Chinese Academy of Sciences, Beijing, China; CAS Key Laboratory of Microbial Physiological and Metabolic Engineering, State Key Laboratory of Microbial Resources, Institute of Microbiology, Chinese Academy of Sciences, Beijing, China; College of Life Science, University of Chinese Academy of Sciences, Beijing, China; CAS Key Laboratory of Microbial Physiological and Metabolic Engineering, State Key Laboratory of Microbial Resources, Institute of Microbiology, Chinese Academy of Sciences, Beijing, China; College of Life Science, University of Chinese Academy of Sciences, Beijing, China

## Abstract

Recent discovery of ectopic repeats (outside CRISPR arrays) provided unprecedented insights into the nondefense roles of CRISPR-Cas. A striking example is the addiction module CreTA (CRISPR-regulated toxin–antitoxins), where one or two (in most cases) ectopic repeats produce CRISPR-resembling antitoxic (CreA) RNAs that direct the CRISPR effector Cascade to transcriptionally repress a toxic RNA (CreT). Here, we demonstrated that CreTA repeats are extensively degenerated in sequence, with the first repeat (ψR1) being more diverged than the second one (ψR2). As a result, such addiction modules become highly specific to their physically-linked CRISPR-Cas loci, and in most cases, CreA could not harness a heterologous CRISPR-Cas to suppress its cognate toxin. We further disclosed that this specificity primarily derives from the degeneration of ψR1, and could generally be altered by modifying this repeat element. We also showed that the degenerated repeats of CreTA were insusceptible to recombination and thus more stable compared to a typical CRISPR array, which could be exploited to develop highly stable CRISPR-based tools. These data illustrated that repeat degeneration (a common feature of ectopic repeats) improves the stability and specificity of CreTA in protecting CRISPR-Cas, which could have contributed to the widespread occurrence and deep diversification of CRISPR systems.

## INTRODUCTION

CRISPR-Cas systems provide prokaryotes with RNA-guided adaptive immunity against viruses and other invasive genetic elements (1,2). The RNA-guided Cas nucleases provide unprecedented specificity and efficiency to recognize a specific nucleic acid sequence, which have revolutionized the genome editing technology (3–5). CRISPR systems are so highly diversified that two classes, six types and 33 subtypes have been defined (6). This diversity provides enormous resources for the innovation of CRISPR-based technologies.

Each CRISPR is an array of identical repeat sequences that are interspaced by invader derived sequences (spacers). Upon virus infection, CRISPR-Cas incorporates a viral DNA fragment into the CRISPR array to record the sequence information of this invader (this process is termed adaptation) (7). Subsequently, CRISPR transcripts were processed by a Cas nuclease (e.g. Cas6 in type I and III systems) or a host enzyme (e.g. RNase III in type II) to give rise to mature CRISPR RNAs (crRNAs), during which the conserved repeats are cleaved and their remnants contribute to the subsequent assembly of a ribonucleoprotein effector (8–11). Based on the complementarity between the spacer and its cognate sequence (protospacer) on the viral DNA/RNA, the effector recognizes and inactivates re-infecting invaders. Though the CRISPR systems are known primarily for its adaptive immunity role, the versatile Cas proteins have been exapted for various nondefense functions (12), which provided illuminating knowledge for developing new CRISPR tools. For example, some degenerated I-B, I-F and V-K CRISPR-Cas loci have been recruited by Tn7-like transposons to achieve targeted transposition (13,14), which were exploited for targeted integration of large DNA fragments into bacterial genomes (15).

The RNA components of CRISPR-Cas systems or, more specifically, their repeat elements have also been extensively exapted for diverse functions that are related or unrelated to viral defence (12). The best-known case is the trans-acting crRNA (tracrRNA) from type II and some type V CRISPR systems, which contains an antirepeat and forms a duplex with one of the repeat sequences of crRNA to facilitate its RNase III-catalyzed maturation (16,17). Another example is the scaRNA (small CRISPR-associated RNA) discovered in *Francisella novicida*, which contains a degenerated repeat and a 15-bp sequence complementary to the 5′ untranslated region of the bacterial lipoprotein operon (18). This complementarity directs Cas9 to transcriptionally repress the lipoprotein genes and regulate the bacterial virulence (19).

**Figure 1.**
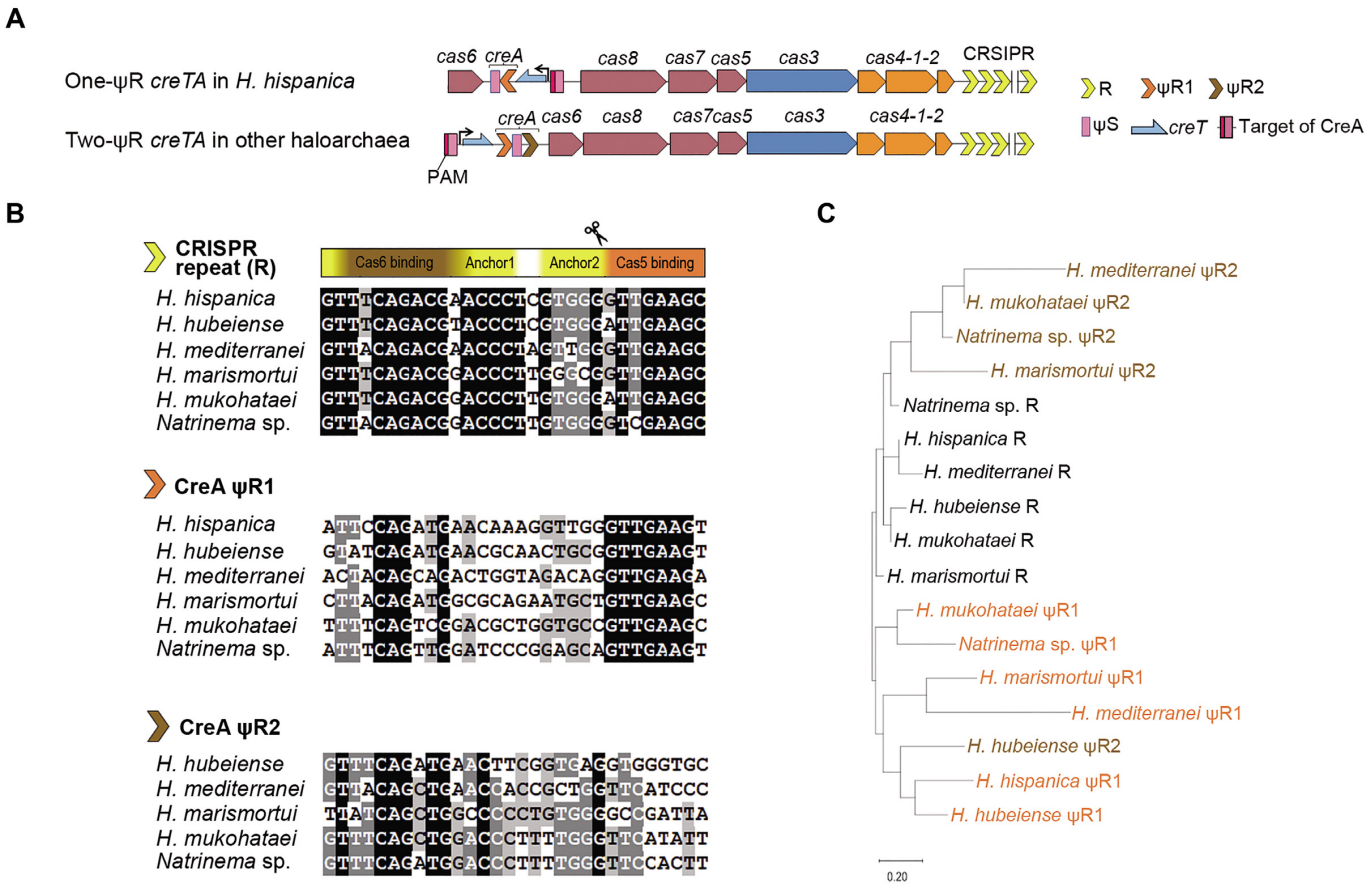
Degeneration of the repeat sequences (ψR1 and ψR2) of haloarchaeal *creTA*. (**A**) Schematic depiction of the *creTA* operons from *H. hispancia* and other haloarchaea. ψS of *creA* partially matches the promoter of *creT*, which is repressed by CreA-guided Cascade. PAM, protospacer adjacent motif (5′-TTC-3′). R, CRISPR repeat. (**B**) Multiple alignment of CRISPR repeat, ψR1 or ψR2 sequences from different haloarchaea. Conserved motifs within CRISPR repeat are indicated, including the conserved trinucleotide at the leader-repeat junction (Junc), the nucleotides critical for Cas6–crRNA binding, the two anchor motifs recognized by the Cas1–Cas2–Cas4 adaptation complex, and the nucleotides critical for Cas5–crRNA binding. The Cas6-processing site on crRNA is indicated by a scissor. (**C**) The phylogenetic tree of CRISPR repeat, ψR1 (orange) and ψR2 (brown) sequences.

Our lab recently reported a distinct form of ‘repeat exaptation’, i.e. the CRISPR-regulated toxin–antitoxin (CreTA) RNA pairs that safeguard type I-B and perhaps other CRISPR-Cas systems (20,21). The *creA* gene contains one or two CRISPR repeat-like sequences (named ΨR) and a spacer-like sequence (ΨS) that is partially complementary to the promoter of the CreT RNA (Figure 1A). This complementarity directs the effector complex Cascade (CRISPR-associated complex for antiviral defence) to transcriptionally repress the toxin gene, which renders the host cell addicted to a functional CRISPR effector and thus ensures robust CRISPR immunity at the population level (once CRISPR-Cas is destroyed, CreT will be liberated to kill the individual host cell). The CreT toxins are highly diverse in sequence and their mechanisms largely remain elusive, except for the cases of *Haloarcula hispanica* and *Halobacterium hubeiense* where CreT acts by sequestering the rare transfer RNA of arginine or isoleucine (20,21). Significantly, CreTA was considered to be the first discovered all-RNA TA module and to represent a unique TA type, i.e. type VIII (22).

Remarkably, contrasting with the conserved repeats in CRISPR arrays, ectopic repeat derivatives all seem to have undergone extensive sequence degeneration or divergence (12,20), of which the biological significance remains unclear. In this study, we focussed on the diverged repeats (ΨR1 and ΨR2) within known CreTA modules, and investigated their roles in deciding the compatibility between CRISPR-Cas and CreTA. Our data showed that CreTA modules have evolved high specificity in safeguarding their respective CRISPR-Cas loci, even though these CRISPR-Cas loci are so closely related and their CRISPR memories are literally interchangeable. Importantly, the safeguarding specificity of CreTA seemed to be driven by the degenerative evolution of its ΨR1 element. Besides, we also showed that the diverged repeats are unlikely to recombine with each other, which could be exploited to engineer highly stable CRISPR arrays for genetic manipulation aims. We conclude that the degenerated repeats of *creTA* modules, on one hand, have laid the foundation for their specificity in protecting the physically linked CRISPR-Cas locus, and on the other, have prevented accidental repeat recombination events that would lead to *creA* inactivation and cell death.

## MATERIALS AND METHODS

### Strains and culture conditions

*H. hispanica* strains used in this study are listed in Supplementary Table S1. The *H. hispanica* ATCC 33960 Δ*pyrF* strain DF60 (uracil auxotrophy) (23) and its derivatives were grown at 37°C in nutrient-rich AS-168 medium (per liter, 200 g NaCl, 20 g MgSO_4_·7H_2_O, 5 g Bacto casamino acids, 5 g yeast extract, 3 g trisodium citrate, 2 g KCl, 50 mg FeSO_4_·7H_2_O, 0.36 mg MnCl_2_·4H_2_O, 1 g sodium glutamate, pH was adjusted to 7.2 with sodium hydroxide) supplemented with uracil at a final concentration of 50 mg/l. Strains transformed with the expression plasmid pWL502 (24) or its derivatives were cultivated in yeast extract-subtracted AS-168 medium, either on solid agar plates or in liquid cultures.

*Escherichia coli* strain DH5α was used for plasmid construction and grown aerobically at 37°C in Luria-Bertani medium. When necessary, ampicillin was added to a final concentration of 100 mg/l.

### Plasmid construction and transformation

The primers used for plasmid engineering in this study are listed in Supplementary Table S2. The double-stranded DNA fragments were amplified using the high-fidelity KOD-Plus DNA polymerase (TOYOBO, Osaka, Japan), then digested with restriction enzymes (New England Biolabs, MA, USA) and ligated into the pWL502 backbone using the T4 DNA ligase (New England Biolabs, MA, USA). Overlap extension polymerase chain reaction (PCR) was conducted as previously described to introduce mutations (21). The constructed plasmids were validated by DNA sequencing and then transformed into the *H. hispanica* strains according to the online Halohandbook (https://haloarchaea.com/wp-content/uploads/2018/10/Halohandbook_2009_v7.3mds.pdf). The yeast extract-subtracted AS-168 plates were used to screen the transformants. The colony forming units per μg plasmid DNA (CFU/μg) were calculated and the log-transformed data were plotted using GraphPad Prism (version 7.00).

### RNA extraction and Northern blot analysis

The *H. hispanica* cells were transformed with crRNA-expressing plasmids. The resulting colonies were then randomly selected and cultured in yeast extract-subtracted AS-168 medium to the exponential phase. After sub-inoculation and cultivation, the exponential phase *H. hispanica* cells were collected by centrifugation and the total RNA was prepared using the TRIzol reagent (Thermo Fisher Scientific, MA, USA) and further purified using the phenol:chloroform method, followed by ethanol precipitation. The RNA samples were quantified using Nanodrop 2000 (Thermo Fisher Scientific, MA, USA). Equal amounts of RNA (ten micrograms) were denatured for 10 min at 65°C with RNA loading dye (New England Biolabs, MA, USA). RNA samples, the Century-Plus RNA ladder (Thermo Fisher Scientific, MA, USA), and the biotin-labeled single-stranded DNA (serving as a custom size marker) were loaded to an 8% polyacrylamide gel (7.6 M urea) and electrophoresed in 1× TBE buffer at 200 V. The RNA ladder lane was excised, stained with Ultra GelRed (Vazyme Biotech, Nanjing, China) and then imaged. After transfer to Biodyne B nylon membrane (Pall, NY, USA) by electroblotting using a Mini-Protean Tetra system (Bio-Rad, CA, USA), the membrane was cross-linked using UV light. The hybridization was performed with biotin-labeled DNA probes. The signals were detected using the Chemiluminescent Nucleic Acid Detection Module Kit (Thermo Fisher Scientific, MA, USA) according to the manufacturer's protocol. The membrane was imaged using the Tanon 5200 Multi chemiluminescent imaging system (Tanon Science & Technology, Shanghai, China).

### qPCR

qPCR was applied to quantify gene expression levels. A total of ten micrograms RNA was treated with RQ1 DNase (Promega, WI, USA) to remove DNA according to the manufacturer's instruction. Then, the pretreated RNA was purified using the phenol:chloroform method and reverse transcribed into complementary DNA (cDNA) using the Moloney Murine Leukemia Virus reverse transcriptase (MMLV-RT) (Promega, WI, USA). qPCR assay was prepared using KAPA SYBR® FAST qPCR Kit (Kapa Biosystems, MA, USA) and performed on an Applied Biosystems ViiA™ 7 Real-Time PCR System. 7*S* RNA gene was used as a loading control. The primers used for qPCR are listed in Supplementary Table S2.

### Virus interference assay

For each transformant of mini-CRISPR-carrying plasmids, individual colonies were randomly picked to inoculate yeast extract-subtracted AS-168 liquid medium. The cultures were then sub-inoculated into fresh medium for another 2-day culturing. 200 μl of cell culture was mixed with 3 ml of molten yeast extract-subtracted AS-168 medium (0.7% top agar) and poured on 1.2% agar plates. After incubation at room temperature for approximately 1 h, 5 μl of 10-fold serial dilutions of the HHPV-2 virus (25) were dropped on top of the *H. hispanica* lawn and dried for approximately 30 min. Then, the plates were incubated for 3–4 days up-side down at 37°C for plaque formation, and individual plaques were counted separately. The ratio of the plaque forming units (PFUs) formed on the empty plasmid-carrying strain divided by the PFUs formed on the mini-CRISPR-carrying strain was used to represent the relative virus immunity (RVI). Three replicates were performed for each condition to get an average and the standard deviation.

### Gene knock-out assay

Plasmids carrying the self-targeting mini-CRISPRs were separately introduced into the wild-type *H. hispanica* cells by transformation. Six replicates were performed for each plasmid and the log-transformed data of transformation efficiency (CFU/μg) were plotted. The number of white and red colonies was also separately recorded. The red surviving colonies were then picked and streaked on a new plate for spacer loss analysis. Colony PCR was performed using the high-fidelity KOD-Plus polymerase to amplify the mini-CRISPR structure, and the PCR products were subjected to Sanger sequencing. The sequencing results were viewed using SnapGene (version 3.2.1).

### Bioinformatic analysis

Repeat sequences were aligned and viewed using the GeneDoc software, and subjected to evolutionary analysis by Maximum Likelihood method using the MEGA software (version 10.1.8). The secondary structure of each repeat sequence was predicted using the RNAfold webserver.

## RESULTS

### ΨR1 is more diverged in sequence from CRISPR repeat than ΨR2

The repeat sequence of a CRISPR is highly compacted with signal nucleotides that are required for crRNA processing, Cascade-crRNA assembly, and adaptation (acquisition of new spacers) (Figure 1B). Early studies on the Cas6 endonuclease demonstrated that it tightly binds to the first 12 nucleotides of the repeat of crRNA, with the primary binding site located within nucleotides 2–8 (8,26), and this tight binding may provide a nucleation core for Cascade assembly (11). In accordance, the first 12 nucleotides of CRISPR repeat are essential for the formation of stable Cascade-crRNA complexes in *H. hispanica* (27). After Cas6-processing, the last 8 nucleotides of the repeat remain on the 5′ end of crRNA (termed 5′ handle) and provide a critical binding site for Cas5 protein during Cascade-crRNA assembly (27–29). As for adaptation, our early study in *H. hispanica* demonstrated that the first 3 nucleotides of the first repeat (immediately downstream of the CRISPR leader) are important, and that nucleotides 11–15 (anchor1; AACCC) and 18–22 (anchor2; GTGGG) essentially involve in dictating the integration sites possibly as two anchors for the adaptation complex (30). To understand the conservation or degeneration of these functional components within haloarchaeal CRISPR repeats or CreTA repeats (ΨR1 and ΨR2), we retrieved these sequences from the six haloarchaeal CRISPR-Cas systems that have been reported to encode a CreTA module (20,21), and subjected them to separate multi-alignment analyses (Figure 1B). As expected, CRISPR repeat holds tight conservation for nucleotides that are critical for any of its three biological functions (adaptation, crRNA processing, and Cascade-crRNA assembly). In comparison, ΨR1 holds more stringent conservation for Cas5-binding nucleotides, but less for Cas6-binding nucleotides, and little to no conservation for the two anchor motifs involved in adaptation (especially for anchor1) (Figure 1B). On the contrary, ΨR2 largely degenerated at the eight Cas5-binding nucleotides, with the other parts being conserved at an intermediate level compared to CRISPR repeat and ΨR1. In total, ΨR1 seemed to be more diverged in sequence from CRISPR repeat. Consistently, on their phylogenetic tree, ΨR2 and CRISPR repeats clustered together, while ΨR1 fell in a separate branch (Figure 1C).

Because many Cas6 nucleases act on the hairpin structures within the CRISPR repeats, we also investigated the hairpin-forming potential of each repeat. Remarkably, we did not observe a hairpin structure that is conserved among the CRISPR repeats, or among the ΨR1 or ΨR2 sequences (Supplementary Figure S1). This result accords with our previous finding that the primary sequence and the secondary structure of haloarchaeal Cas6 both exhibit more similarity to *Pyrococcus furiosus* Cas6 (PfCas6), which adopts a wrap-around mechanism in processing an unstructured RNA substrate, than to Cas6 proteins that recognize the structural elements within repeat RNA (10). We surmise that haloarchaeal Cas6 nucleases do not recognize structural elements within the repeats of CRISPR or CreTA, and hence impose no structural constraints on their evolution.

### *H. hispanica* CRISPR-Cas cannot regulate the CreTA of four closely-related systems

**Figure 2.**
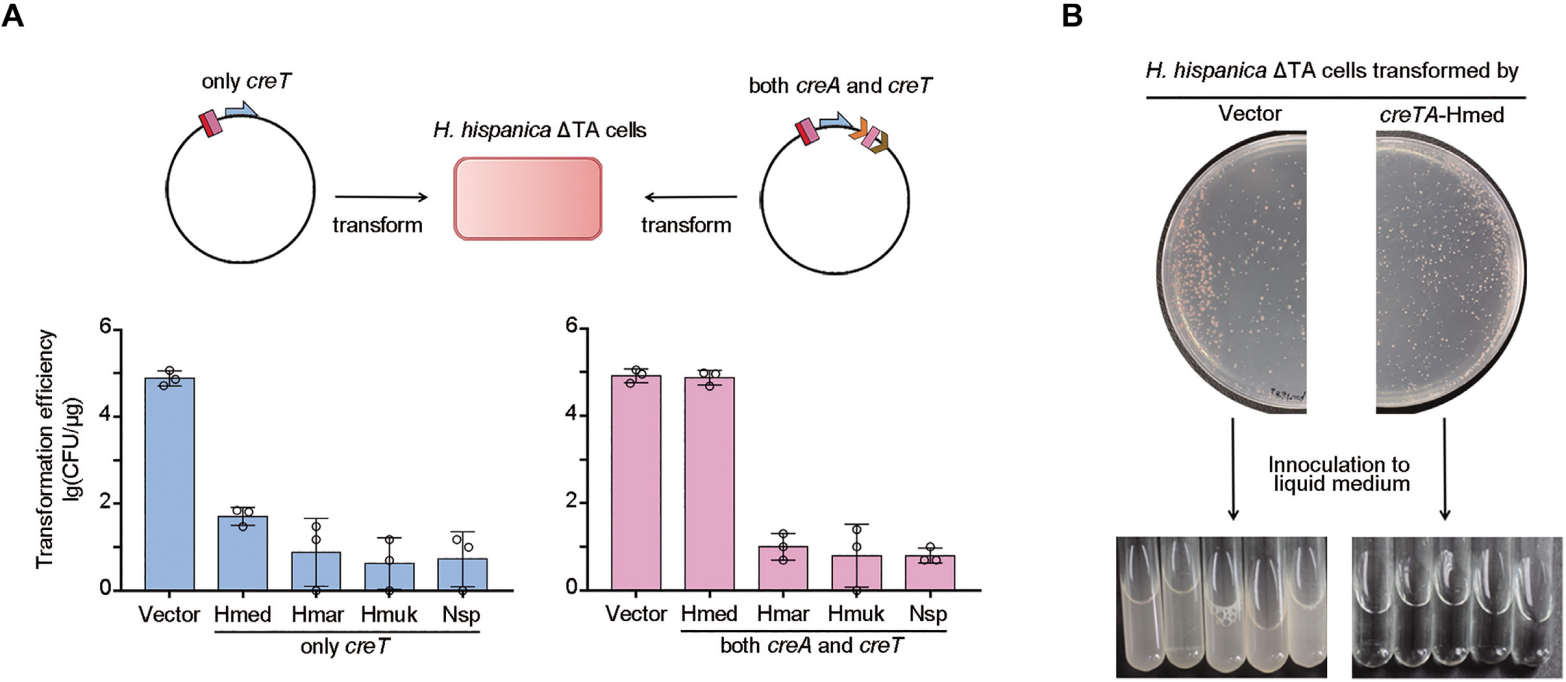
*H. hispanica* Cascade cannot fully repress the toxicity of four heterologous *creTA* modules. (**A**) Transformation of *H. hispanica* cells lacking endogenous *creTA* (ΔTA) with a plasmid carrying only *creT*, or both *creT* and *creA*, of other haloarchaea. Hmed, *H. mediterranei*; Hmar, *H. marismortui*; Hmuk, *H. mukohataei*; Nsp, *Natrinema* sp. CFU, colony forming units. Error bars, mean ± s.d. (*n* = 3). (**B**) When transformed by a plasmid carrying *H. mediterranei creTA*, *H. hispanica* ΔTA cells formed tiny colonies on plates and could not grow in liquid culture.

Though our latest study showed that the *H. hubeiense* CreTA could be heterologously regulated by *H. hispanica* CRISPR-Cas (20), we still doubted whether CRISPR-Cas systems could commonly share their ‘addiction’ module just like sharing the CRISPR memory among closely-related systems, regarding that ΨR1 and ΨR2 hold less conservation for the Cas6-binding nucleotides compared to CRISPR repeat (Figure 1B). Therefore, in a *H. hispanica* mutant devoid of its own *creTA* (ΔTA), we tested the activity of the toxin (*creT*) and the antitoxin (*creA*) moieties of the CRISPR ‘addiction’ modules from other haloarchaea. Compared to the empty vector, derivates carrying the *creT* gene from *Haloferax mediterranei*, *Halomicrobium mukohataei*, *Haloarcula marismortui*, or *Natrinema* sp. J7-2, consistently showed a marked reduction (by ∼10^4^ fold) in transformation efficiency (Figure 2A), indicating these toxin genes were all functional in the heterologous host. When their respective *creA* genes were also included in the recombinant plasmids, the transformation efficiency, however, was still markedly reduced (by ∼10^4^ fold) compared to the empty vector, except in the case of *H. mediterranei creTA* (Figure 2A). It was suggested that *H. mukohataei*, *H. marismortui*, and *Natrinema* sp. *creA* genes could not repress their cognate *creT* with the help of *H. hispanica* CRISPR-Cas.

Though the plasmid carrying *H. mediterranei creTA* transformed *H. hispanica* ΔTA cells with a high efficiency that is equivalent to the empty vector, we noticed that the transformants formed colonies that were smaller in size compared to those containing the empty vector, and importantly, they could not grow in liquid culture (Figure 2B). It appears that *H. mediterranei creA* only partially suppressed the toxicity of *creT*. Then we introduced this plasmid into *H. hispanica* ΔTAΔ*cas6* cells that lack the nuclease required for CreA maturation (21). Surprisingly, we still observed a high transformation efficiency (equivalent to the empty vector), and again, the transformants could not grow in liquid culture (Supplementary Figure S2). Therefore, the observed partial suppression of *H. mediterranei creT* could not be attributed to the joint effect of *H. hispanica* Cascade and mature *H. mediterranei* CreA RNAs. In another word, like the case of *H. mukohataei*, *H. marismortui*, or *Natrinema* sp., *H. mediterranei creA* could neither harness *H. hispanica* CRISPR-Cas for toxin repression.

### *H. hispanica* Cas6 cannot process the diverged repeats of heterologous CreTA

**Figure 3.**
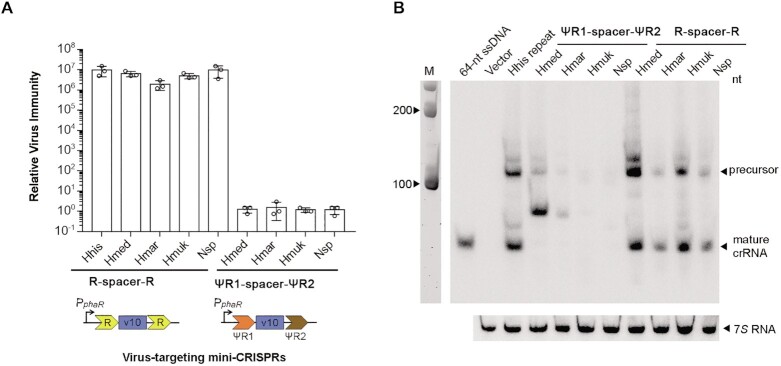
Immunity effects (**A**) and Northern blotting analysis (**B**) of crRNAs with different repeat sequences in *H. hispanica* ΔTA cells. The v10 spacer targeting HHPV-2 was designed between two CRISPR repeats from different haloarchaea or between their ψR1 and ψR2 sequences. A strong promoter (P*_phaR_*) was used to drive the transcription of these mini-CRISPRs. The relative virus immunity was calculated by dividing the plaque forming units (PFUs) on cells containing the empty vector (pWL502) by the PFUs on cells containing a crRNA-expressing plasmid. Error bars, mean ± s.d. (*n* = 3). Cells containing the empty vector were used as the negative control (Vector) in the blotting assay. 7*S* RNA served as the internal control. A biotin-labeled 64-nt ssDNA was co-electrophoresed. M, 100-nt ssRNA ladder.

We inferred that *H. hispanica* Cas6 could not process these noncognate CreA RNAs due to their degenerated repeats (ΨR1 and ΨR2). To test this possibility, we engineered a series of mini-CRISPRs, where the v10 spacer (targeting the HHPV-2 virus of *H. hispanica* (25,27)) was sandwiched by two identical CRISPR repeats or by the degenerated ΨR1 and ΨR2 sequences from different haloarchaea (Figure 3A), and introduced them into *H. hispanica* ΔTA cells. By Northern blotting, we found that mini-CRISPRs containing CRISPR repeats from different haloarchaea consistently produced mature v10 crRNAs (64 nt), which were of more abundance when the CRISPR repeat of *H. hispanica*, *H. mukohataei*, or *H. mediterranei* was utilized (Figure 3B). In sharp contrast, mature v10 crRNAs were not detected for any constructs with ΨR1 and ΨR2. Then we subjected the transformed *H. hispanica* cells to HHPV-2 infection, and observed that mini-CRISPRs with the conserved CRISPR repeats caused robust virus immunity, i.e. a ∼10^7^-fold reduction in plaque forming units (Figure 3A). However, as expected, mini-CRISPRs constructed with ΨR1 and ΨR2 provided little to no immunity. Hence, we conclude that, through artificial (or natural) gene transfer events, the haloarchaeal CRISPR-Cas systems could share invader information with their CRISPR repeats holding tight sequence conservation, while in most cases, they could not share their ‘addiction’ modules where the repeat elements are highly degenerated.

The Northern blotting analysis, interestingly, showed that the mini-CRISPR containing *H. mediterranei* ΨR1 and ΨR2 produced an unexpected RNA product, estimated to be ∼80 nt (Figure 3B). Notably, in a host lacking the Cas6 nuclease, this RNA product could still be detected (Supplementary Figure S3B), indicating that its production did not rely on Cas6 catalysis. We surmise that a similar Cas6-independent small RNA may produce from the wild *creA* gene of *H. mediterranei* and perhaps account for the partial *creT* suppression observed in ΔTA (Figure 2) and ΔTAΔ*cas6* (Supplementary Figure S2) cells.

### Specificity between CRISPR-Cas and CreTA is primarily caused by ΨR1 degeneration

**Figure 4.**
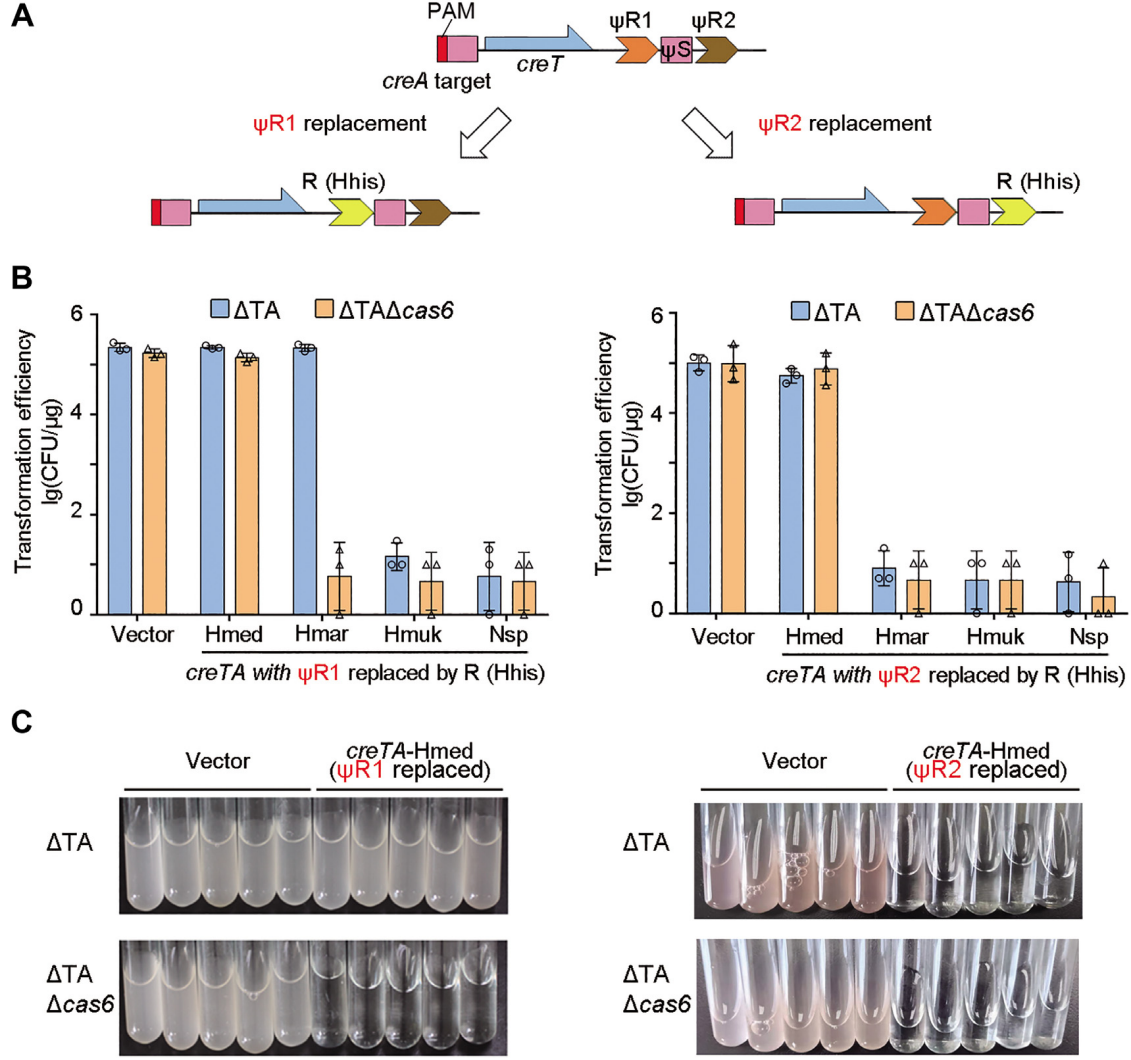
*H. hispanica* Cascade repressed *H. mediterranei* and *H. marismortui creTA* modules when their ψR1, rather than ψR2, was replaced by the CRISPR repeat of *H. hispanica*. (**A**) Scheme illustrating the separate replacement of ψR1 and ψR2. (**B**) Transformation of *H. hispanica* ΔTA or ΔTAΔ*cas6* cells by plasmids carrying a repeat-replaced *creTA* module. Error bars, mean ± s.d. (*n* = 3). (**C**) Growth of *H. hispanica* ΔTA cells in liquid AS-168 medium (yeast extract-subtracted).

Then we tested the possibility of altering the specificity between CRISPR-Cas and CreTA by repeat replacement. To adapt the heterologous CreTA modules to *H. hispanica* CRISPR-Cas, we separately replaced their ΨR1 and ΨR2 sequences with the CRISPR repeat of *H. hispanica* (Figure 4A). Because ΨR1 appears to be more diverged from CRISPR repeat than ΨR2 (Figure 1), we predicted that replacing ΨR1 may bring about more compatibility between *creA* and Cas6. As expected, *H. marismortui creTA* showed toxicity (∼10^4^-fold reduction in transformation efficiency) in both ΔTA and ΔTAΔ*cas6* cells of *H. hispanica* when its ΨR2 was replaced, but ΨR1 replacement recovered the transformation efficiency of ΔTA cells, not that of ΔTAΔ*cas6* cells, to the level of the vector control (Figure 4B). This indicated that *H. marismortui* CreA harnessed *H. hispanica* CRISPR-Cas to suppress its cognate toxin after its ΨR1 rather than ΨR2 was altered to the CRISPR repeat of *H. hispanica*.

In line with the partial toxin repression observed for the wild *H. mediterranei* CreTA (Figure 2; Supplementary Figure S2), plasmids carrying the ΨR1- or ΨR2-replaced *H. mediterranei creTA* transformed both ΔTA and ΔTAΔ*cas6* cells with a high efficiency that was equivalent to the empty vector (Figure 4B). But notably, when we inoculated the transformants into liquid culture, only ΔTA cells containing the ΨR1-replaced *creTA* could grow (Figure 4C). It was indicated that *H. mediterranei* CreA fully repressed its cognate toxin in *H. hispanica* after its ΨR1 rather than ΨR2 was replaced, which coincided with the effects of repeat replacement on *H. marismortui* CreTA. Therefore, we conclude that specificity between CRISPR-Cas and CreTA is primarily driven by ΨR1 degeneration.

**Figure 5.**
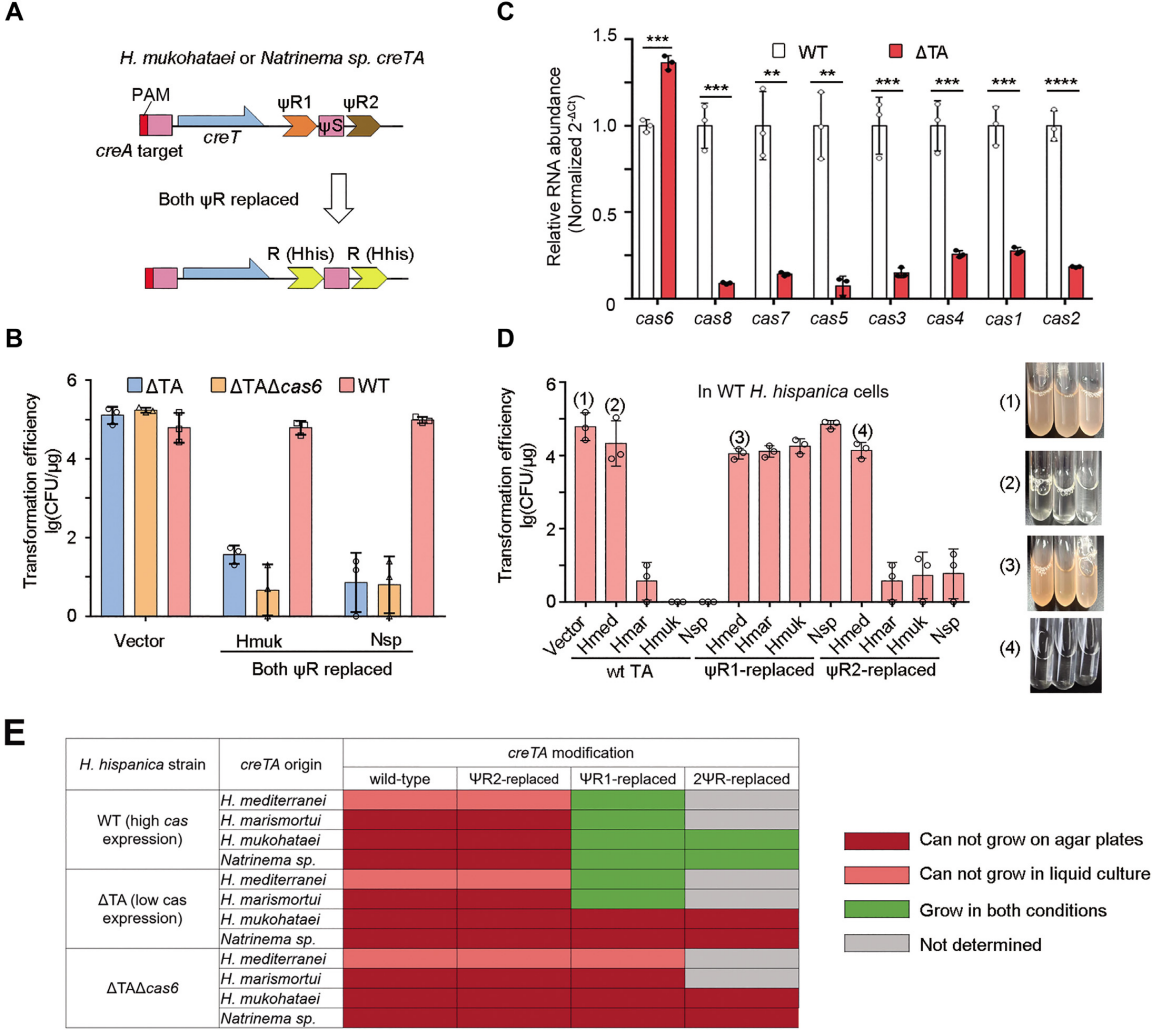
Outcomes of CRISPR-CreTA interplay in different strains of *H. hispanica*. (**A**) Simultaneous replacing of the ψR1 and ψR2 elements of *H. mukohataei* or *Natrinema* sp. *creTA* with the CRISPR repeat (R) of *H. hispanica* (Hhis). (**B**) Transformation of *H. hispanica* ΔTA, ΔTAΔ*cas6*, or WT cells by a plasmid carrying both-ψR-replaced *creTA* modules. (**C**) RNA abundance was shown as 2^–ΔCt^ (normalized for WT) for each *cas* gene relative to the internal control 7*S* RNA. Error bars, mean ± s.d. (*n* = 3). (**D**) Transformation of *H. hispanica* WT cells by a plasmid carrying the wild-type (wt), ψR1-replaced, or ψR2-replaced *creTA* module. For each of the four indicated plasmids, three individual colonies were selected to test their growth in liquid culture. (**E**) Growth of *H. hispancia* cells with different *cas* backgrounds and various *creTA* elements.

However, interestingly, recovery of transformation efficiency was not observed for plasmids carrying *H. mukohataei* or *Natrinema* sp. *creTA* when either ΨR1 or ΨR2 was replaced (Figure 4B). Then we simultaneously replaced their ΨR1 and ΨR2 with *H. hispanica* CRISPR repeat, but again, the carrying plasmids still transformed ΔTA cells with markedly reduced efficiency compared to the empty vector (Figure 5B). It seemed that, aside from the degenerated repeats of CreTA, other factors should also affect the outcome during its interplay with CRISPR-Cas.

### The expression level of Cas proteins may influence the outcome of CRISPR-TA interplay

To explore such factors, we introduced the both-ΨR-replaced *creTA* elements of *H. mukohataei* and *Natrinema* sp. into more *H. hispanica* strains. Unexpectedly, the carrying plasmids showed high transformation efficiency (∼10^5^ CFU/μg; equivalent to the empty vector) in the WT *H. hispanica* cells (the native *creTA* was not deleted) (Figure 5B). It seemed that, under this genetic background, the toxicity of these heterologous *creTA* elements became fully suppressed, which contrasted with the results in ΔTA cells. We considered that this discrepancy should derive from potential polar effects of *creTA* deletion, rather than from the native *creTA per se*, because CreA transcriptionally represses *creT* based on the complementarity between its ΨS and the promoter DNA of *creT* and the haloarchaeal *creA* genes studied here carry distinct ΨS sequences (21). So, we probed the transcription level of each *cas* gene separately in WT versus in ΔTA cells (Figure 5C). Remarkably, when the *cas6-cas8* intergenic region containing *creTA* was absent (in ΔTA), transcription of *cas8*, *cas7*, *cas5*, *cas3*, *cas4*, *cas1* and *cas2* was reduced by 91.1%, 85.9%, 92.6%, 85.0%, 74.2%, 72.4% and 81.6%, respectively; by contrast, *cas6* transcription appeared to be slightly up-regulated (by ∼36.5%). These results demonstrated that a strong promoter has evolved within the *cas6-cas8* intergenic region and largely promoted the transcription of the downstream *cas* genes. Note that mature v10-crRNAs were still not detected in WT *H. hispanica* cells when the mini-CRISPRs were constructed with heterologous *creTA* repeats (Supplementary Figure S3C), suggesting the compatibility between Cas6 nuclease and repeat elements should be the key factor deciding CreTA specificity.

With the above knowledge, we rechecked the compatibility between *H. hispanica* CRISPR-Cas and each modified or unmodified *creTA* element in WT cells. Consistent to the results in ΔTA cells, plasmids carrying the wild-type or the ΨR2-replaced *H. mukohataei*, *H. marismortui*, and *Natrinema* sp. *creTA* all transformed WT *H. hispanica* cells with markedly reduced efficiency compared to the empty vector (Figure 5D). Besides, WT cells containing *H. mediterranei creTA* could grow in liquid culture when ΨR1 was replaced (by the CRISPR repeat of *H. hispancia*), but not when ΨR2 was replaced, which also accorded with the results in ΔTA cells. Nevertheless, different from the results in ΔTA cells (Figure 4B), recovery of high-level transformation efficiency (10^4^–10^5^ CFU/μg) by ΨR1 replacement was observed not only for *H. marismortui creTA*, but also for *H. mukohataei* and *Natrinema* sp. *creTA* in WT cells (Figure 5D). These results supported that the interplay between CRISPR-Cas and CreTA could give rise to different outcomes in strains with differing *cas* expression levels (summarized in Figure 5E), and on the other hand confirmed our conclusion that the CRISPR-TA specificity derives mainly from the degenerative evolution of the first repeat element (ΨR1) of *creA*.

### Exploiting degenerated CreTA repeats to stabilize engineered CRISPRs

**Figure 6.**
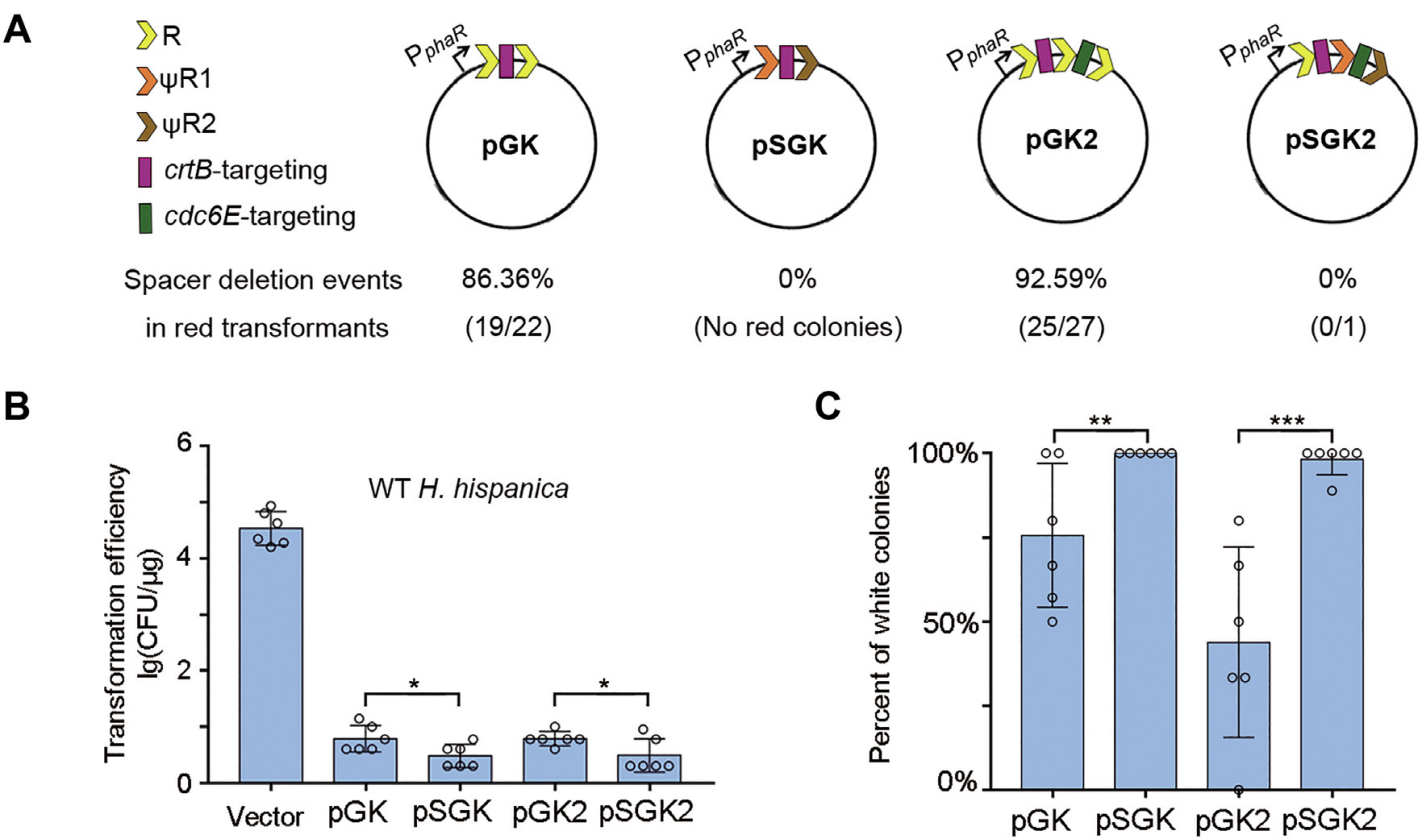
Exploiting *creTA* repeats to improve the stability and efficiency of a CRISPR tool. (**A**) The design of mini-CRISPRs with or without degenerated repeats (ψR1 and ψR2). Two spacers were designed to target non-essential genes, *crtB* (required for producing red pigments) and *cdc6E* (a dispensable DNA replication-related gene), respectively. (**B**) Transformation of *H. hispanica* WT cells by the plasmids carrying a self-targeting CRISPR. (**C**) The percent of white colonies formed in pGK, pSGK, pGK2, or pSGK2 transformation assays. Error bars, mean ± s.d. (*n* = 6); one-tailed Student's *t* test (*P< 0.05, **P < 0.01, ***P < 0.001).

The invader information stored in a CRISPR array is continuingly updated through acquisition of new spacers and deletion of existing spacers, the latter of which involves the recombination events between two of the identical CRISPR repeats. However, when we employ CRISPR-Cas for genetic manipulation, CRISPR variability or instability is unfavoured, especially when more than one target gene is simultaneously manipulated (i.e. multiplexing). We expected that the degenerated repeat elements of *creTA* may be exploited to circumvent this issue. In our previous study, we reprogrammed the native CRISPR-Cas of *H. hispanica* with a gene knock-out plasmid (pGK; see Figure 6A) to remove the *crtB* gene (HAH_2563) that involves in carotenoid synthesis and confers a red colour on colonies (31). Here, we replaced the two CRISPR repeats (flanking the *crtB*-targeting spacer) with the degenerated repeats (ΨR1 and ΨR2) from *H. hubeiense creTA* (which is naturally compatible with *H. hispanica* CRISPR-Cas (20)), to generate a stable gene knock-out plasmid, namely pSGK (Figure 6A). Then we introduced pGK and pSGK separately into WT *H. hispanica* cells without providing a repair donor. In principle, CRISPR-mediated cleavage of chromosome killed most cells and only a small fraction that lost or mutate the target gene could survive and form white colonies (the *crtB*^–^ phenotype). Though these two plasmids both showed a transformation efficiency that was ∼10^4^-fold reduced relative to the empty vector, pSGK did result in fewer transformants than pGK (*P*= 0.019; one-tailed *t* test) (Figure 6B). Notably, all the colonies obtained from six duplicates of the pSGK transformation assay exhibited a white colour (the *crtB*^–^ phenotype), while ∼25% of the pGK colonies remained in red (Figure 6C). We then inspected the pGK plasmid in these red colonies by DNA sequencing and found that the *crtB*-targeting spacer lost via repeat recombination at a frequency of 86.36% (19/22) (Figure 6A and Supplementary Figure S4).

By including another spacer targeting *cdc6E* (HAH_1857; a redundant replication-related gene) (31), we further constructed pGK2 and pSGK2 to test the advantage of exploiting *creTA* repeats for multiplexing (Figure 6A). Note that, within the mini-CRISPR of pSGK2, CRISPR repeat (of *H. hispanica*), ΨR1 and ΨR2 (of *H. hubeiense*) were designed as the first, second and third repeat elements, respectively. As expected, pSGK2 showed a lower efficiency than pGK2 when transforming WT *H. hispanica* cells (*P*= 0.024; one-tailed *t* test) (Figure 6B), and white colonies accounted for ∼44% and ∼98% during pGK2 and pSGK2 transformation assays, respectively (Figure 6C). Among the red colonies of pGK2, the two spacers lost together at a frequency of 92.59% (25/27) (Figure 6A and Supplementary Figure S4). In fact, from six duplicates of the pSGK2 transformation assay, we only obtained a single red colony, and remarkably, its mini-CRISPR stubbornly remained intact (Supplementary Figure S4), indicating that this colony survived CRISPR targeting through mechanisms other than spacer loss. So we conclude that, replacing the conserved CRISPR repeats with the degenerated *creTA* repeats can generally eliminates the spacer loss risk of CRISPR tools. This finding also implies that the degenerative evolution of *creTA* repeats have substantially prevented repeat recombination that may cause ΨS deletion and *creT*-induced cell death.

## DISCUSSION

From the early days of CRISPR research, this prokaryotic defense system has been repeatedly proposed to commit nondefense functions such as regulating the expression of bacterial genes (32–34). However, experimental evidences supporting these proposals have been scarce until the recent discovery of some functional ectopic repeats (outside CRISPR arrays) that give rise to crRNA-like small RNAs. The scaRNA characterized in *F. novicida* repurposes the catalytic-active Cas9 (of type II, Class 2) to repress the transcription of the bacterial lipoprotein operon, which is required for bacterial virulence (19). Similarly, the CreA RNAs discovered in haloarchaea repurposed the type I-B (of Class 1) effector Cascade to transcriptionally repress an RNA toxin gene (*creT*) without recruiting the Cas3 nuclease for DNA cleavage, and this regulatory circuit renders the host cell addicted to the immunity effector (20,21). Hence, it seems currently to be a general paradigm for different CRISPR types that the catalytic-active CRISPR-Cas apparatus is employed by microbes for simultaneous viral defense and gene regulation, and notably, the latter function could make host cells addicted to CRISPR defense directly as an addiction module (like the case of CreTA) or indirectly by conferring fitness advantages (like the case of scaRNA).

**Figure 7.**
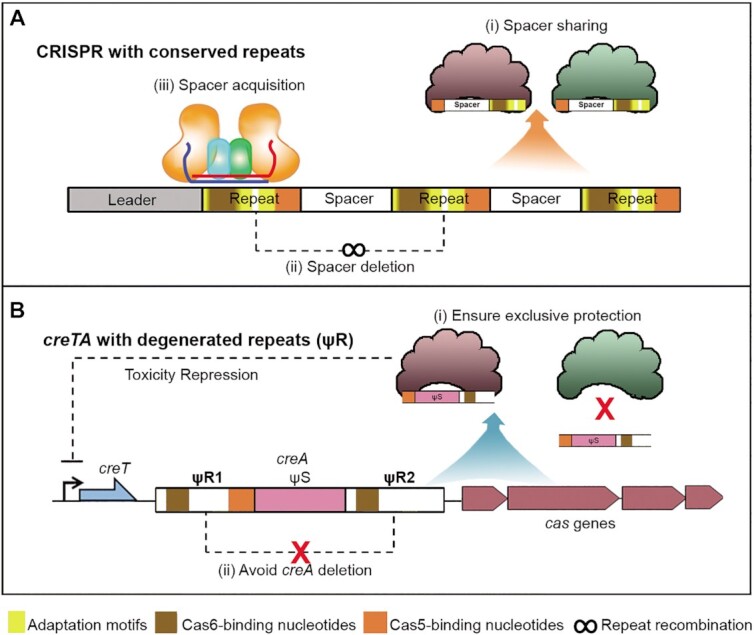
Model illustrating the biological significances of the conserved CRISPR repeats and the degenerated CreTA repeats. (**A**) The conservation of CRISPR repeats facilitates or supports the sharing of invader information among closely related systems (i), the repeat recombination process that removes existing spacers (ii), and the CRISPR adaptation process that incorporates new spacers (iii). (**B**) The degeneration of CreTA repeats ensures the specificity of CreTA in protecting CRISPR-Cas (i) and avoids *creA* corruption via recombination events (ii). Conserved nucleotides involving in CRISPR adaptation, Cas6 binding, and Cas5 binding, are indicated with yellow, brown and orange blocks, respectively.

Remarkably, a common feature of these ectopic repeats is that they all have undergone extensive degeneration compared to the conserved CRISPR repeats (19,20). In this study, we systemically investigated the biological significance of repeat degeneration of the haloarchaeal CreTA ‘addiction’ modules. Our data demonstrated that the repeats (more specifically, ΨR1) of different CreTA modules have evolved to be so diverged in sequence that they cannot be processed by the Cas6 nuclease from a very closely related CRISPR system, and hence rendered these addiction modules exclusive to their physically-linked and coevolving CRISPR-Cas loci (Figure 7B). In contrast, the haloarchaeal CRISPR arrays hold tight conservation in repeat sequence and could share their invader information among related systems (Figure 7A), which will certainly improve their ability to defeat common invaders. We also illustrated that degenerated *creA* repeats are much more stable than the conserved CRISPR repeats because the latter are prone to recombination, which in fact contributes to the updating of CRISPR memory (Figure 7A). While in the case of CreTA, its insusceptibility to repeat recombination avoids *creA* corruption and accidental cell death (Figure 7B). Our bioinformatic analysis also illustrated that the essential nucleotides involved in new spacer acquisition are strictly conserved among CRISPR repeats, but became highly diverged among *creA* repeats (particularly among the ΨR1 sequences) (Figure 1). Therefore, we propose that the degeneration of ectopic repeats is an adaptation to their nondefense functions, which require less ‘spacer’ dynamics and more interacting specificity with CRISPR-Cas so that they could render the host cell addicted more exclusively to the CRISPR-Cas locus coevolving with them.

Our data also showed that the interplay between CRISPR-Cas and CreTA could give rise to different outcomes depending on the *cas* background (Figure 5E). Regardless of the requirements for repeat replacement, it seemed that, to repress the toxicity of some CreTA modules (like those from *H. mukohataei* and *Natrinema* sp.), a higher level of Cascade proteins was required compared to the other modules (like CreTA from *H. marismortui* and *H. mediterranei*). This finding implies that the CreTA addiction module will be triggered to induce cell dormancy or death when a certain portion (not all) of the cellular pool of CRISPR effectors get inactivated by anti-CRISPR proteins. We surmise that other factors, like the complementarity between *creA* and its target sequence, the promoter strength of the toxin gene, etc., may also influence the outcome during CRISPR-CreTA interplay, and these factors should have been fine-tuned by selection during CreTA evolution.

In summary, though the ectopic repeats could be diverse in their regulating targets and biological functions, it may be their common feature to undergo degeneration, by which they get more stable and more specific to the coevolving CRISPR-Cas locus that has spawned them. These ‘locus-specific’ ectopic repeats, which could confer ‘CRISPR addiction’ or fitness advantages on the host cell, should play important roles during the wide spread and deep diversification of CRISPR-Cas systems.

## DATA AVAILABILITY

All data are available in the manuscript and the supplementary data.

## Supplementary Material

gkac712_Supplemental_File
