# Supplementary material for: The toxin–antitoxin RNA guards of CRISPR-Cas evolved high specificity through repeat degeneration
Source: Nucleic Acids Res. 2022 Aug 26;50(16):9442–52. doi: 10.1093/nar/gkac712 (PMC9458426; doi:10.1093/nar/gkac712)
Supplement: gkac712_Supplemental_File [file gkac712_supplemental_file.pdf]

Supplementary material for：

The toxin-antitoxin RNA guards of CRISPR-Cas evolved high specificity through repeat degeneration

Feiyue Cheng[1], Aici Wu[1,2], Chao Liu[1,2], Xifeng Cao[3], Rui Wang[1], Xian Shu[1,2], Lingyun Wang[1,4], Yihan Zhang[1,3], Hua Xiang[1,2,*] and Ming Li[1,2,*]

[1]CAS Key Laboratory of Microbial Physiological and Metabolic Engineering, State Key Laboratory of Microbial Resources, Institute of Microbiology, Chinese Academy of Sciences, Beijing, China.
[2]College of Life Science, University of Chinese Academy of Sciences, Beijing, China.
[3]School of life Sciences, Hebei University, Baoding, Hebei, China.
[4]College of Plant Protection, Shandong Agricultural University, Taian, Shandong, China.

* To whom correspondence should be addressed. Tel: 010-64807064; Fax: 010-64807064; Email: lim_im@im.ac.cn; Correspondence can also be addressed to Hua Xiang. Email: xiangh@im.ac.cn

The file includes:

Figures S1 to S4

Tables S1 to S2

References

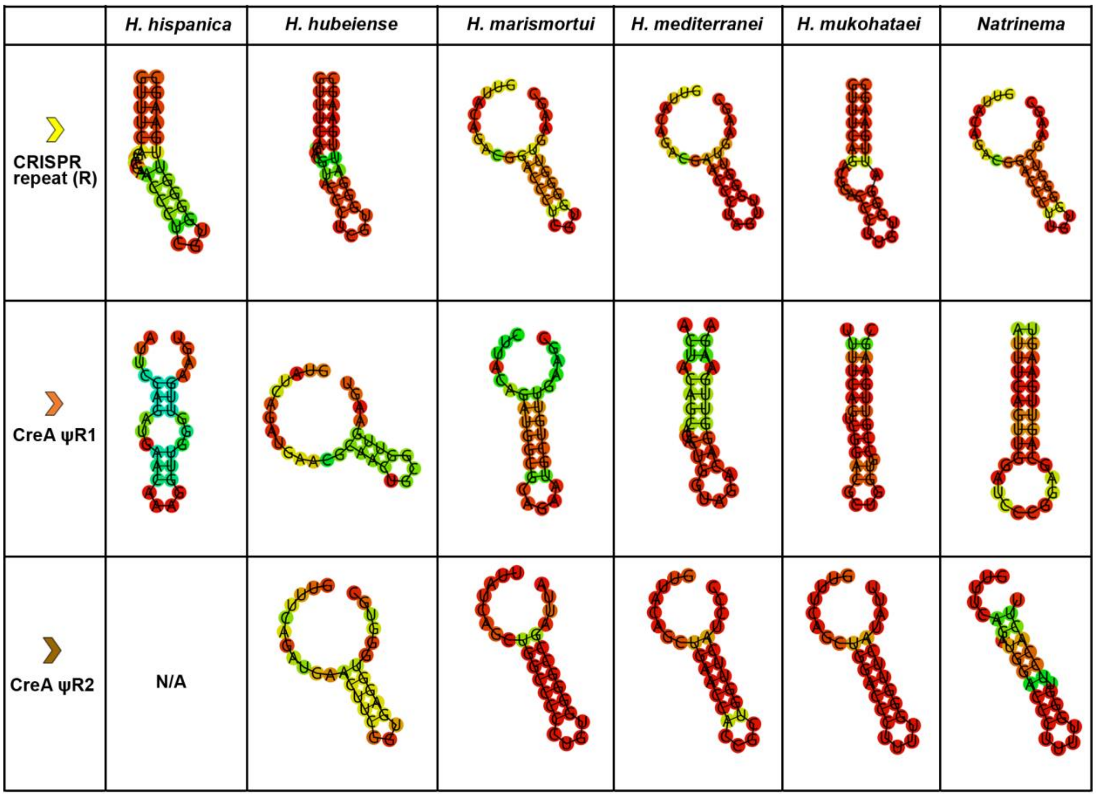

Figure S1 The secondary structure of CRISPR repeat (R) and ΨR sequences (ΨR1 and ΨR2) from different haloarchaea. The RNAfold webserver (1) was used to predict the folding potential of different repeat sequences.

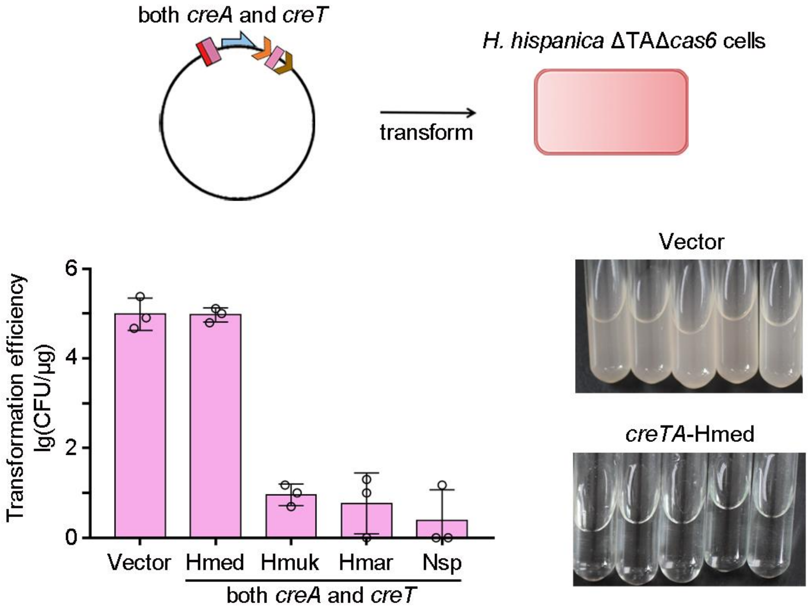

Figure S2 Transformation of *H. hispanica* cells lacking the Cas6 nuclease with a plasmid expressing a heterologous *creTA*. For the plasmid carrying *H. mediterranei creTA,* five individual colonies were randomly selected and inoculated into the liquid medium. Error bars, mean ± s.d. (n = 3).

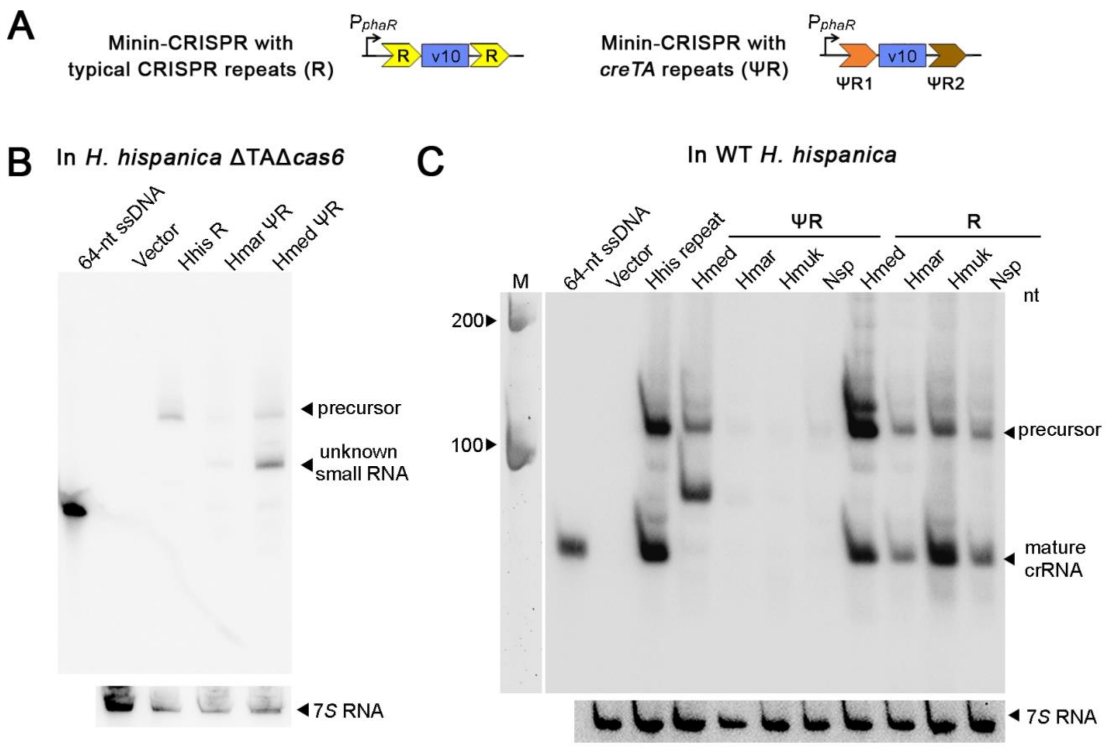

Figure S3 Northern blotting analysis of crRNAs with different repeat sequences in *H. hispanica* cells. (A) Illustration of the crRNA-expressing plasmids. P_{phaR}, a strong constitutive promoter, was used to drive the transcription of mini-CRISPRs. The v10 spacer targeting the HHPV-2 virus was sandwiched by two CRISPR repeats or by ΨR sequences (ΨR1 and ΨR2). (B) Northern blotting of the RNA products from mini-CRISPRs in *H. hispanica* cells lacking its native *creTA* and Cas6 nuclease. (C) Blotting of crRNAs in the wild-type (WT) strain. The expected size of mature crRNA was 64-nt, and a biotin-labeled synthetic ssDNA of this size was co-electrophoresed. 7*S* RNA was probed as the internal control. M, 100-nt RNA marker.

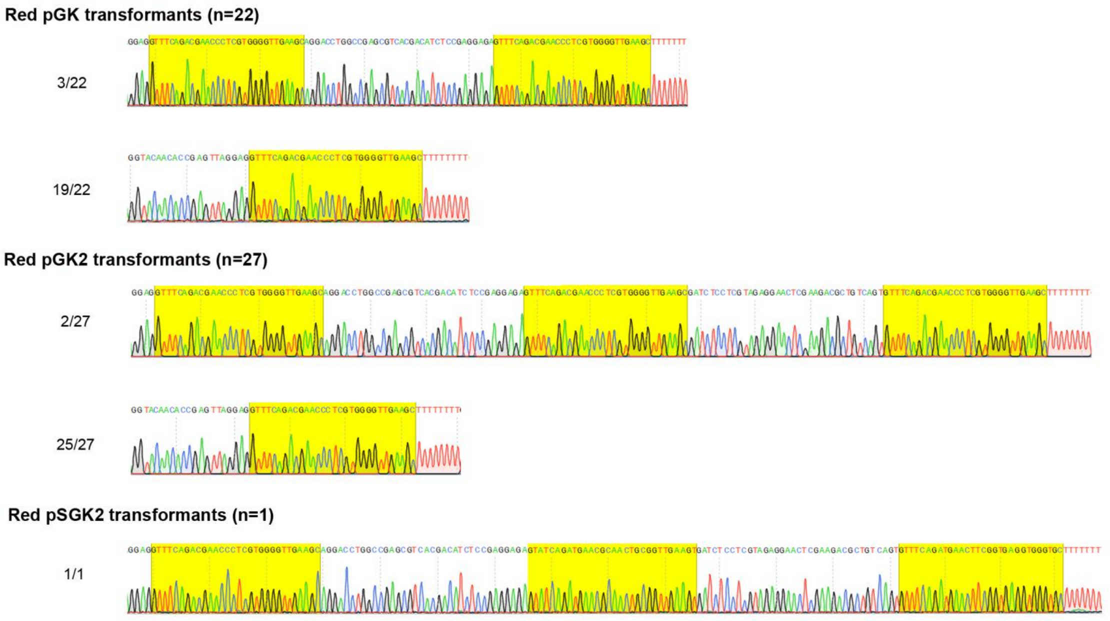

Figure S4 Sequencing results of the gene-knockout plasmids in the red survivors. Plasmids carrying a single-spacer CRISPR targeting *crtB* (pGK and pSGK) or a two-spacer CRISPR simultaneously targeting *crtB* and *cdc6* (pSGK and pSGK2) were transformed into the WT *H. hispanica* cells, and surviving colonies of red color (*crtB*$^{+}$ phenotype) were subjected to PCR analysis and DNA sequencing. The representative sequencing results were shown. Repeat sequences are indicated in yellow.

Table S1 Strains used in this study

Strain Name	Description	References
WT (DF60)	*pyrF* deletion mutant of *H. hispanica* ATCC 33960	(2)
ΔTA	*creTA* deletion mutant of DF60	(3)
ΔTAΔ*cas6*	*cas6* deletion mutant of ΔTA	

Table S2 Oligonucleotides used in this study

Name	Sequence (5´-3´)[a]	Description
**For amplification of *creTA* analogs**		
Hmed-TAL-F	CGCGGATCCAACCCCCGGGGGGTCGA	Forward primer for *H. mediterranei creTA* amplification
Hmed-TAL-R	CGGGGTACCTGACTGTTCTCTGTAGGA	Reverse primer for *H. mediterranei creTA* amplification
Hmar-TAL-F	CGCGGATCCGACATCCTCGACCACGAA	Forward primer for *H. marismortui creTA* amplification
Hmar-TAL-R	CGGGGTACCCCGATTACATCACCTCGA	Reverse primer for *H. marismortui creTA* amplification
Hmuk-TAL-F	CGCGGATCCCGGACGGTTTACTGGGGT	Forward primer for *H. mukohataei creTA* amplification
Hmuk-TAL-R	CGGGGTACCCCGTTGTACTCCCTACTT	Reverse primer for *H. mukohataei creTA* amplification
Nsp7-TAL-F	CATGCCATGGTGATGAAGACCAAAGGAC	Forward primer for Natrinema *creTA* amplification
Nsp7-TAL-R	CGGGGTACCTTACCGATTGCATCATGATA	Reverse primer for Natrinema *creTA* amplification
**For amplification of ψR-replaced *creTA***		
Hmed-ψR-R	TTGGACTTCCATCTAAGGCA	Mutating ψR
Hmed-ψR1-F	TGCCTTAGATGGAAGTCCAAGTTTCAGACGAACCCTCGTGGGGTTGAAGCATACACCACGCTAGGTGGCT	mutating the ψR1 to *H. hispanica* CRISPR repeat
Hmar-ψR-R	TACCTATTTTGTAGGGACAG	Mutating ψR
Hmar-ψR1-F	CTGTCCCTACAAAATAGGTAGTTTCAGACGAACCCTCGTGGGGTTGAAGCAGCTACCCGTGTTACTCGGG	mutating the ψR1 to *H. hispanica* CRISPR repeat
Hmuk-ψR-R	TTCGACTGTACTACGGGCAT	Mutating ψR
Hmuk-ψR1-F	ATGCCCGTAGTACAGTCGAAGTTTCAGACGAACCCTCGTGGGGTTGAAGCATGTTCGAAGCTGTTGCGGG	mutating the ψR1 to *H. hispanica* CRISPR repeat
Nsp-ψR-R	CGATCTGTACTGCGGGCATG	Mutating ψR
Nsp-ψR1-F	CATGCCCGCAGTACAGATCGGTTTCAGACGAACCCTCGTGGGGTTGAAGCTTGTTCGGACCCG	mutating the ψR1 to *H. hispanica*

	CTGTGGG	CRISPR repeat
Hmed-ψR2-R1	AATGCTTCAACCCCACGAGGGTTCGTCTGAAACGTACCTTAGAATTAAGCC	mutating the ψR2 to *H. hispanica* CRISPR repeat
Hmed-ψR2-R2	CGGGGTACCTGACTGTTCTCTGTAGGAGTATATAAATTCTAACGTGTCTGAAAATGCTTCAACCCCACGAGGG	mutating the ψR2 to *H. hispanica* CRISPR repeat
Hmar-ψR2-R	AAGGCTTCAACCCCACGAGGGTTCGTCTGAAACCAGGTCCCTACGAGTTCC	mutating the ψR2 to *H. hispanica* CRISPR repeat
Hmar-ψR2-F	AACCCTCGTGGGGTTGAAGCCTTTCACTGCGCTAGAC	mutating the ψR2 to *H. hispanica* CRISPR repeat
Hmuk-ψR2-R	ATGGCTTCAACCCCACGAGGGTTCGTCTGAAACAGCAGTGCCCGCAACAGC	mutating the ψR2 to *H. hispanica* CRISPR repeat
Hmuk-ψR2-F	AACCCTCGTGGGGTTGAAGCCATCATCACCACGATTAC	mutating the ψR2 to *H. hispanica* CRISPR repeat
Nsp-ψR2-R	TGAGCTTCAACCCCACGAGGGTTCGTCTGAAACTAGCTTTGGCCCACAGCG	mutating the ψR2 to *H. hispanica* CRISPR repeat
Nsp-ψR2-R1	CGGGGTACCTTACCGATTGCATCATGATAAATGTGATTGTGCTAGAGTGAGCTTCAACCCCACGAGGGTTC	mutating the ψR2 to *H. hispanica* CRISPR repeat
Nsp-ψR12-R	CGGGGTACCTTACCGATTGCATCATGATAAATGTGATTGTGCTAGAGTGA	mutating the ψR1 and ψR2 to CRISPR repeat
Nsp-ψR12-R1	ATGTGATTGTGCTAGAGTGAGCTTCAACCCCACGAGGGTTCGTCTGAAACTAGCTTTGGCCCACAGCGGG	mutating the ψR1 and ψR2 to *H. hispanica* CRISPR repeat
Nsp-ψR12-R2	ATGTGATTGTGCTAGAGTGAGCTTCAACCCCACGAGGGTCCGTCTGTAACTAGCTTTGGCCCACAGCGGG	mutating the ψR1 and ψR2 to the CRISPR repeat of *H. hispanica* and that of *H. marismortui*
Hmuk-ψR12-R	CGGGGTACCCCGTTGTACTCCCTACTTCGTGTCATTTTGTGTCATTGTGATACTCACAACCGAACGATTCTAT	mutating the ψR1 and ψR2 to CRISPR repeat
Hmuk-ψR12-R1	ACTCACAACCGAACGATTCTATATAAATGTAATCGTGGTGATGATGGCTTCAACCCCACGAGGGTTCGTCTGAAACAGCAGTGCCCGCA	mutating the ψR1 and ψR2 to the CRISPR repeat of *H. hispanica*

Hmuk-ψR12-R2	ACAACCGAACGATTCTATATAAATGTAATCGTGGTGATGATGGCTTCAACCCCACGAGGGTCCGTCTGTAACAGCAGTGCCCGCAACAG	mutating the ψR1 and ψR2 to the CRISPR repeat of *H. hispanica* and that of *H. marismortui*
**For construction of mini-CRISPR**		
Hmed-ψR-V10-F	CGCGGATCCCGAAGGGAACATATATGTTACTGCAGGTACAACACCGAGTTAGGAGACTACAGCAGACTGGTAGACAGGTT	Contains the sequence of P_{phaR}
Hmed-ψR-V10-R	GTAACTCGCTATACCCGGCCCCCGTCGTGGTCTGCGAAATCTTCAACCTGTCTACCAGTCTGCT	amplifying the artificial mini-CRISPR array
Hmed-ψR-V10-R1	CGGGGTACCAAAAAAAAGGGATGAACCAGCGGTGGTTCAGCTGTAACTCGCTATACCCGGCC	amplifying the artificial mini-CRISPR array
Hmar-ψR-V10-F	CGCGGATCCCGAAGGGAACATATATGTTACTGCAGGTACAACACCGAGTTAGGAGCTTACAGATGGCGCAGAATGCTGTT	amplifying the artificial mini-CRISPR array
Hmar-ψR-V10-R	TGATAATCGCTATACCCGGCCCCCGTCGTGGTCTGCGAAAGCTTCAACAGCATTCTGCGCCATC	amplifying the artificial mini-CRISPR array
Hmar-ψR-V10-R1	CGGGGTACCAAAAAAAATAATCGGCCCCACAGGGGGCCAGCTGATAATCGCTATACCCGGCC	amplifying the artificial mini-CRISPR array
Hmuk-ψR-V10-F	CGCGGATCCCGAAGGGAACATATATGTTACTGCAGGTACAACACCGAGTTAGGAGTTTTCAGTCGGACGCTGGTGCCGTT	amplifying the artificial mini-CRISPR array
Hmuk-ψR-V10-R	TGAAACTCGCTATACCCGGCCCCCGTCGTGGTCTGCGAAAGCTTCAACGGCACCAGCGTCCGAC	amplifying the artificial mini-CRISPR array
Hmuk-ψR-V10-R1	CGGGGTACCAAAAAAAAAATATGAACCCAAAAGGGTCCAGCTGAAACTCGCTATACCCGGCC	amplifying the artificial mini-CRISPR array
Nsp-ψR-V10-F	CATGCCATGGCGAAGGGAACATATATGTTACTGCAGGTACAACACCGAGTTAGGAGATTTCAGTTGGATCCCGGAGCAGTT	amplifying the artificial mini-CRISPR array
Nsp-ψR-V10-R	TGAAACTCGCTATACCCGGCCCCCGTCGTGGTCTGCGAAAACTTCAACTGCTCCGGGATCCAAC	amplifying the artificial mini-CRISPR array
Nsp-ψR-V10-R1	CGGGGTACCAAAAAAAAAAGTGGAACCCAAAAGGGTCCATCTGAAACTCGCTATACCCGGCC	amplifying the artificial mini-CRISPR array
phaR-F	CGCGGATCCCGAAGGGAACATATATGTTACTGCAGGTACAACACCGAGTTAGGAG	Contains the sequence of P_{phaR}

crtB-R-R1	TCTCCTCGGAGATGTCGTGACGCTCGGCCAGGTCCTGCTTCAACCCCACGAGGGTTCGTCTGAAACCTCCTAACTCGGTGTTGTAC	amplifying the *crtB*-targeting CRISPR
crtB-R-R2	CGGGGTACCAAAAAAAAGCTTCAACCCCACGAGGGTTCGTCTGAAACTCTCCTCGGAGATGTCGTGA	amplifying the *crtB*-targeting CRISPR
crtB-ψR-R1	TCTCCTCGGAGATGTCGTGACGCTCGGCCAGGTCCTACTTCAACCGCAGTTGCGTTCATCTGATACCTCCTAACTCGGTGTTGTAC	amplifying the *crtB*-targeting CRISPR
crtB-ψR-R2	CGGGGTACCAAAAAAAAGCACCCACCTCACCGAAGTTCATCTGAAACTCTCCTCGGAGATGTCGTGA	amplifying the *crtB*-targeting CRISPR
crtBcdc6-R-R1	TCTTCGAGTTCCTCTACGAGGAGATCGCTTCAACCCCACGAGGGTTCGTCTGAAACTCTCCTCGGAGATG	amplifying the *crtB*-*cdc6E*-targeting CRISPR
crtBcdc6-R-R2	CGGGGTACCAAAAAAAAGCTTCAACCCCACGAGGGTTCGTCTGAAACACTGACAGCGTCTTCGAGTTCCTCTACGAGGAGATC	amplifying the *crtB*-*cdc6E*-targeting CRISPR
crtBcdc6-ψR-R1	AGCGTCTTCGAGTTCCTCTACGAGGAGATCACTTCAACCGCAGTTGCGTTCATCTGATACTCTCCTCGGAGATGTCGTGA	amplifying the *crtB*-*cdc6E*-targeting CRISPR
crtBcdc6-ψR-R2	CGGGGTACCAAAAAAAAGCACCCACCTCACCGAAGTTCATCTGAAACACTGACAGCGTCTTCGAGTTCCTCTA	amplifying the *crtB*-*cdc6E*-targeting CRISPR
**For qPCR analysis**		
cas1-F	ACAGTCAACACCGACGACAA	against the sequence of *cas1* gene
cas1-R	GTCGCCTCGATACCCATCAG	against the sequence of *cas1* gene
cas2-F	GCTCAAAATTGGTCGGCGAT	against the sequence of *cas2* gene
cas2-R	CGTAGACTGTCCGGTTGAGC	against the sequence of *cas2* gene
cas3-F	TATCACGCTCCAATCGGCTC	against the sequence of *cas3* gene
cas3-R	TCTGAGTTGTACGCACGGTC	against the sequence of *cas3* gene

cas4-F	TGACCGATGCAGATCCAGTC	against the sequence of *cas4* gene
cas4-R	AGGCTGTCTCATCAACACGG	against the sequence of *cas4* gene
cas5-F	CAATCGCTGGACAGCTGGA	against the sequence of *cas5* gene
cas5-R	CGACAGTGTGTTCATCGGCA	against the sequence of *cas5* gene
cas6-F	GCACGGATGCAGCTTATCA	against the sequence of *cas6* gene
cas6-R	CGTGACGTCGTCAACGCGAA	against the sequence of *cas6* gene
cas7-F	CAGTTGACCCCGTAACGAAC	against the sequence of *cas7* gene
cas7-R	CAAGATCGTAGTCGTCGGGA	against the sequence of *cas7* gene
cas8-F	CAGAGAAATTCCGCGAGGAGT	against the sequence of *cas8* gene
cas8-R	CTGAATCAGGTCGTCAGTGTA	against the sequence of *cas8* gene
7S-F	TCGATGGTCCGCTGCTCAC	against the sequence of 7*S* RNA gene
7S-R	ACCGGGGGCGTCCGGTCTGA	against the sequence of 7*S* RNA gene
**For DNA sequencing**		
test-F	TCGGGTATCTCGTAATCTCGT	against the sequence of empty vector
test-R	GAGTGCGACGACCGTATGTA	against the sequence of empty vector
**For Northern blotting**		

Hhis-7S-probe	CGTCAGACCGGACGCCCCC	biotin-labeled; for Northern analysis
v10-probe	CCGTCGTGGTCTGCGAAA	biotin-labeled; for Northern analysis

[a]underlined, restriction sites; F, forward primer; R, reverse primer; double underlined, overlapping sequences designed for overlap extension PCR.

REFERENCE

1. Lorenz,R., Bernhart,S.H., Siederdissen,C.H.Z., Tafer,H., Flamm,C., Stadler,P.F. and Hofacker,I.L. (2011) ViennaRNA Package 2.0. *Algorithm Mol. Biol.*, **6**, 26.
2. Liu,H.L., Han,J., Liu,X.Q., Zhou,J. and Xiang,H. (2011) Development of *pyrF*-based gene knockout systems for genome-wide manipulation of the archaea *Haloferax mediterranei* and *Haloarcula hispanica*. *J. Genet. Genomics*, **38**, 261-269.
3. Li,M., Gong,L.Y., Cheng,F.Y., Yu,H.Y., Zhao,D.H., Wang,R., Wang,T., Zhang,S.J., Zhou,J., Shmakov,S.A. *et al.* (2021) Toxin-antitoxin RNA pairs safeguard CRISPR-Cas systems. *Science*, **372**, eabe5601.
